# Mortality-associated Risk Factors in Hospitalized COVID-19 Patients in Japan: Findings of the CLOT-COVID Study

**DOI:** 10.2188/jea.JE20220201

**Published:** 2023-03-05

**Authors:** Makoto Takeyama, Sen Yachi, Yuji Nishimoto, Ichizo Tsujino, Junichi Nakamura, Naoto Yamamoto, Hiroko Nakata, Satoshi Ikeda, Michihisa Umetsu, Shizu Aikawa, Hiroya Hayashi, Hirono Satokawa, Yoshinori Okuno, Eriko Iwata, Yoshito Ogihara, Nobutaka Ikeda, Akane Kondo, Takehisa Iwai, Norikazu Yamada, Tomohiro Ogawa, Takao Kobayashi, Makoto Mo, Yugo Yamashita

**Affiliations:** 1 Japan Community Health Care Organization Tokyo Shinjuku Medical Center, Tokyo, Japan; 2 Hyogo Prefectural Amagasaki General Medical Center, Osaka, Japan; 3 Hokkaido University Hospital, Sapporo, Japan; 4 Hamamatsu Medical Center, Shizuoka, Japan; 5 Yokosuka General Hospital Uwamachi, Kanagawa, Japan; 6 Nagasaki University Graduate School of Biomedical Sciences, Nagasaki, Japan; 7 Tohoku University Hospital, Sendai, Japan; 8 Tsukuba Medical Center Hospital, Ibaraki, Japan; 9 Osaka Metropolitan University Graduate School of Medicine, Osaka, Japan; 10 Fukushima Medical University, School of Medicine, Fukushima, Japan; 11 Kyoto University Hospital, Kyoto, Japan; 12 Nankai Medical Center Japan Community Health Care Organization, Oita, Japan; 13 Mie University Hospital, Tsu, Japan; 14 Toho University Ohashi Medical Center, Tokyo, Japan; 15 Shikoku Medical Center for Children and Adults, Kagawa, Japan; 16 Tsukuba Vascular Center, Ibaraki, Japan; 17 Kuwana City Medical Center, Mie, Japan; 18 Fukushima Daiichi Hospital, Fukushima, Japan; 19 Yokohama Minami Kyosai Hospital, Yokohama, Japan

**Keywords:** COVID-19, mortality, risk factors, thrombosis

## Abstract

**Background:**

Reports of mortality-associated risk factors in patients with the novel coronavirus disease 2019 (COVID-19) are limited.

**Methods:**

We evaluated the clinical features that were associated with mortality among patients who died during hospitalization (*n* = 158) and those who were alive at discharge (*n* = 2,736) from the large-scale, multicenter, retrospective, observational cohort CLOT-COVID study, which enrolled consecutively hospitalized COVID-19 patients from 16 centers in Japan from April to September 2021. Data from 2,894 hospitalized COVID-19 participants of the CLOT-COVID study were analyzed in this study.

**Results:**

Patients who died were older (71.1 years vs 51.6 years, *P* < 0.001), had higher median D-dimer values on admission (1.7 µg/mL vs 0.8 µg/mL, *P* < 0.001), and had more comorbidities. On admission, the patients who died had more severe COVID-19 than did those who survived (mild: 16% vs 63%, moderate: 47% vs 31%, and severe: 37% vs 6.2%, *P* < 0.001). In patients who died, the incidence of thrombosis and major bleeding during hospitalization was significantly higher than that in those who survived (thrombosis: 8.2% vs 1.5%, *P* < 0.001; major bleeding: 12.7% vs 1.4%, *P* < 0.001). Multivariable logistic regression analysis revealed that age >70 years, high D-dimer values on admission, heart disease, active cancer, higher COVID-19 severity on admission, and development of major bleeding during hospitalization were independently associated with a higher mortality risk.

**Conclusion:**

This large-scale observational study in Japan identified several independent risk factors for mortality in hospitalized patients with COVID-19 that could facilitate appropriate risk stratification of patients with COVID-19.

## INTRODUCTION

The novel coronavirus disease 2019 (COVID-19) is an enormous global threat^1^^,^^2^ that has affected more than 517 million people and has caused more than 6.2 million deaths as of May 2022.^3^ Appropriate mortality risk stratification is essential for the clinical identification of patients who require close monitoring and intensive treatment. Several clinical studies have reported mortality-associated risk factors in COVID-19 patients worldwide, including in North America, Europe, and China.^3^^–^^13^ Recent meta-analyses have shown that advanced age; male sex; current smoking; and comorbidities, such as diabetes, hypertension, asthma, heart disease, obesity, and active cancer are independent risk factors for COVID-19 mortality.^14^^,^^15^

Nonetheless, the clinical features of COVID-19 patients and the mortality risk could vary by race or ethnicity, clinical practice, and accessibility of medical resources in each country and region. A meta-analysis from overseas showed that the mortality rate of hospitalized patients with COVID-19 was 17.6%,^15^ while the corresponding rate in Japan was 5.7%.^16^ A few studies have reported the risk factors for COVID-19 “severity” in Japanese patients; these include a study that analyzed data in a nationwide registry.^17^ However, there have been no reports on the risk factors for COVID-19-associated mortality in Japanese patients hospitalized for the disease; therefore, our large-scale, multicenter, observational study aimed to investigate such risk factors.

## METHODS

### Study population

This physician-initiated, observational cohort study involved the analysis of data accumulated in the nationwide “The Thrombosis and Anticoagulation Therapy in patients with COVID-19 in Japan Study” (CLOT-COVID Study; UMIN000045800), which enrolled consecutive hospitalized COVID-19 patients from April to September 2021 in 16 centers in Japan.^18^^–^^22^ The primary objective of the CLOT-COVID study was to reveal the current status of thrombosis and anticoagulation therapy in patients with COVID-19 in Japan.^18^ Patients with COVID-19 confirmed by polymerase chain reaction were eligible for inclusion. The CLOT-COVID Study enrolled 2,894 hospitalized COVID-19 patients. The study population was divided into patients who died during hospitalization and those who survived to discharge. We compared the patient characteristics during hospitalization and the clinical outcomes between the two groups and investigated the risk factors for mortality.

We performed all study-related procedures in accordance with the code of medical ethics specified in the Declaration of Helsinki. All participating centers’ ethics committees or relevant review boards approved the research protocol. The Fukushima Daiichi Hospital’s ethical committee served as the primary ethics committee (Approval number: 2021-11-2). The requirement of written informed consent was waived because we used clinical data obtained in routine clinical practice and none of the patients refused to participate when contacted for follow-up. By posting detailed information about the studies on each institution’s website, the patients could request to opt out of the study at any time between enrollment and follow-up. This method is concordant with the guidelines for epidemiological studies issued by the Ministry of Health, Labour, and Welfare in Japan.

### Data collection and definition of participant characteristics

Based on prespecified definitions, we searched the hospital databases and accumulated data regarding the participants’ clinicodemographic characteristics, pharmacological thromboprophylaxis management, and clinical outcomes. These data were entered into an electronic case report form. At the general office, the data were checked for missing inputs and values that were outside the anticipated range.

We classified the severity of COVID-19 as mild, moderate, and severe if the patients did not need oxygen, needed oxygen, or needed mechanical ventilation or extracorporeal membrane oxygenation, respectively.^23^^,^^24^ We defined pharmacological thromboprophylaxis as the use of anticoagulants during hospitalization, not including use for the treatment of thrombosis. We included patients who continued anticoagulant therapy from prior to hospitalization. Therapeutic unfractionated heparin use was definded as the use of unfractionated heparin while aming for a therapeutic range based on the activated partial thromboplastin time (APTT). We defined prophylactic unfractionated heparin use as the use of unfractionated heparin without consideration of APTT. eMaterial 1 contains detailed definitions of the other participant characteristics.

### Clinical outcomes

We evaluated the occurrence of thrombotic events, major bleeding, and all-cause mortality during hospitalization as clinical outcomes. The primary endpoint was all-cause mortality, and the secondary endpoints were thrombosis and major bleeding. In this study, we defined venous thromboembolism (VTE), ischemic stroke, myocardial infarction, and systemic arterial thromboembolism as thrombosis. VTE included deep vein thrombosis or pulmonary embolism diagnosed by ultrasonography, contrast-enhanced computed tomography, pulmonary angiography, ventilation-perfusion lung scintigraphy, contrast venography, or autopsy. Ischemic stroke was defined as a stroke that lasted longer than 24 hours and necessitated or extended hospitalization. We defined myocardial infarction based on the universal myocardial infarction guidelines.^25^ Finally, major bleeding was defined by a minimum 2 g/dL reduction in the hemoglobin level, transfusion of at least two units of blood, or hemorrhage at a critical site according to the International Society of Thrombosis and Hemostasis guidelines.^26^

### Statistical analysis

Categorical variables are summarized as the number and percentage and were compared using Fisher’s exact test or the chi-square test as appropriate. Continuous variables are expressed, based on their distribution, as the mean and standard deviation or the median and interquartile range, and they were compared using Student’s *t*-test or the Wilcoxon rank-sum test. The number of events and the percentages of clinical outcomes are presented. Furthermore, using stratified analysis, we evaluated the clinical outcomes based on COVID-19 severity at the time of admission. To investigate the mortality-associated risk factors during hospitalization, we performed multivariable logistic regression analysis to estimate the odds ratios (ORs) and 95% confidence intervals (Cis) of the potential risk factors. In accordance with previous reports^14^^,^^15^^,^^27^^–^^29^ and with regard to clinical relevance, we selected 13 clinically relevant variables from among the baseline characteristics (age >70 years, male sex, body mass index >30 kg/m^2^, D-dimer values on admission >1.0 µg/mL, hypertension, diabetes mellitus, heart disease, respiratory disease, active cancer, history of major bleeding, history of VTE, and moderate and high COVID-19 severity at admission), the use of pharmacological thromboprophylaxis, and the development of thrombosis and major bleeding during hospitalization. Patients were considered to have missing data if data regarding the D-dimer values quantified at the time of admission were missing. We performed a multivariable logistic regression analysis after excluding the missing data. R version 4.1.2 was used for all statistical analyses (R Foundation for Statistical Computing, Vienna, Austria).^30^ All tests were two-tailed, and statistical significance was set at *P* < 0.05.

## RESULTS

### Participant characteristics

**Figure 1.**
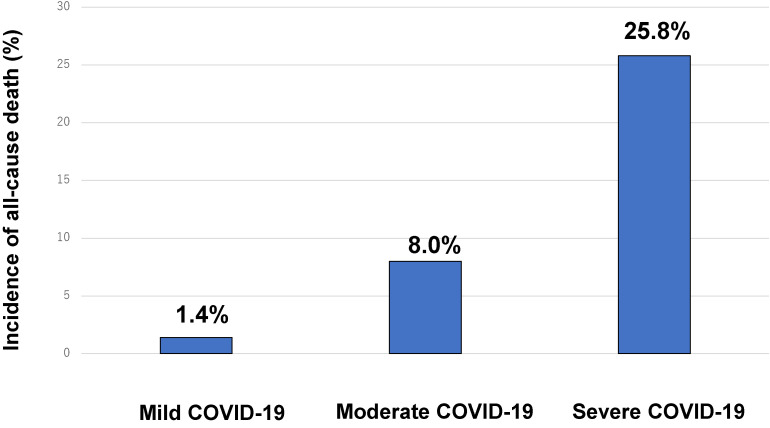
Incidence of all-cause mortality according to the COVID-19 severity on admission. Patients with mild, moderate, and severe COVID-19 were defined as those who did not require oxygen, required oxygen, and required mechanical ventilation or extracorporeal membrane oxygenation. COVID-19, novel coronavirus disease 2019.

Among the 2,894 hospitalized COVID-19 patients, 158 died during hospitalization (hospital mortality 5.5%). A higher COVID-19 severity on admission was associated with an increase in the in-hospital mortality (mild 1.4%; moderate 8.0%; and severe 25.8%; Figure 1). Patients who died during hospitalization were older (71.1 years vs 51.6 years, *P* < 0.001), had higher median D-dimer values on admission (1.7 µg/mL vs 0.8 µg/mL, *P* < 0.001), and had more comorbidities, including hypertension (56% vs 29%, *P* < 0.001), diabetes mellitus (30% vs 20%, *P* = 0.006), heart disease (25% vs 7.9%, *P* < 0.001), active cancer (8.2% vs 1.7%, *P* < 0.001), and history of VTE (1.9% vs 0.4%, *P* = 0.045; Table 1). In patients who died, the median hospitalization duration was longer than that in those who survived (16 vs 9 days, *P* < 0.001). In the group of patients who died, a higher proportion of patients used pharmacological thromboprophylaxis than in the group of those who survived (82% vs 41%, *P* < 0.001).

**Table 1.** Patient characteristics and pharmacological thromboprophylaxis managements

	Total (*N* = 2,894)	Patients who died during hospitalization (*N* = 158)	Patients alive at discharge (*N* = 2,736)	*P*-value
**Baseline characteristics**				
Age, years	52.7 (17.9)	71.1 (11.8)	51.6 (17.6)	<0.001
Age >70 years	535 (19%)	99 (63%)	436 (16%)	<0.001
Men	1,885 (65%)	109 (69%)	1,776 (65%)	0.35
Body weight, kg	68.9 (18.5)	66.0 (16.1)	69.0 (18.6)	0.049
Height, cm	164.4 (12.4)	163.1 (9.8)	164.4 (12.5)	0.19
Body mass index, kg/m^2^	25.3 (5.4)	24.7 (4.9)	25.3 (5.4)	0.18
Body mass index ≥30 kg/m^2^	459 (16%)	20 (13%)	439 (16%)	0.31
D-dimer level on admission, µg/mL (*N* = 2,771)	0.8 (0.5–1.3)	1.7 (1.1–3.5)	0.8 (0.5–1.2)	<0.001
D-dimer level on admission >1.0 µg/mL (*N* = 2,771)	974 (35%)	120 (80%)	854 (32%)	<0.001
Length of hospitalization, days	9 (6–14)	16 (10–24)	9 (6–13)	<0.001
**Comorbidities**				
Hypertension	874 (30%)	89 (56%)	785 (29%)	<0.001
Diabetes mellitus	597 (21%)	47 (30%)	550 (20%)	0.006
Heart disease	255 (8.8%)	40 (25%)	215 (7.9%)	<0.001
Respiratory disease	298 (10%)	21 (13%)	277 (10%)	0.22
Active cancer	60 (2.1%)	13 (8.2%)	47 (1.7%)	<0.001
History of major bleeding	28 (1.0%)	4 (2.5%)	24 (0.9%)	0.06
History of VTE	15 (0.5%)	3 (1.9%)	12 (0.4%)	0.045
**Severity of COVID-19 on admission**				
Mild	1,738 (60%)	25 (16%)	1,713 (63%)	<0.001
Moderate (Need oxygen)	927 (32%)	74 (47%)	853 (31%)	
Severe (Need mechanical ventilation/ECMO)	229 (7.9%)	59 (37%)	170 (6.2%)	
**Pharmacological thromboprophylaxis managements**				
Anticoagulants	1,245 (43%)	130 (82%)	1,115 (41%)	<0.001
Unfractionated heparin of prophylactic dose	685/1,245 (55%)	44/130 (34%)	641/1,115 (58%)	<0.001
Unfractionated heparin of therapeutic dose	161/1,245 (13%)	55/130 (42%)	106/1,115 (9.5%)	
Low-molecular-weight heparin of prophylactic dose	204/1,245 (16%)	16/130 (12%)	188/1,115 (17%)	
Low-molecular-weight heparin of therapeutic dose	0/1,245 (0%)	0/130 (0%)	0/1,115 (0%)	
Direct oral anticoagulants	164/1,245 (13%)	14/130 (11%)	150/1,115 (14%)	
Warfarin	19/1,245 (1.5%)	1/130 (0.8%)	18/1,115 (1.6%)	
Others	12/1,245 (1.0%)	0/130 (0%)	7/1,115 (0.6%)	

APTT, activated partial thromboplastin time; COVID-19, novel coronavirus disease 2019; ECMO, extracorporeal membrane oxygenation; VTE, venous thromboembolism.

Categorical variables are presented as numbers and percentages, and continuous variables are presented as the mean and standard deviation or the median and interquartile range based on their distributions. Categorical variables were compared using the chi-squared test when appropriate; otherwise, Fisher’s exact test was used. Continuous variables were compared using the Student’s *t* test or Wilcoxon’s rank sum test based on distribution. Unfractionated heparin of a therapeutic dose was defined as the administration of unfractionated heparin targeting a therapeutic range referencing the APTT. Unfractionated heparin of a prophylactic dose was defined as the administration of unfractionated heparin of a fixed dose without a referencing the APTT.

### Clinical outcomes during hospitalization

**Figure 2.**
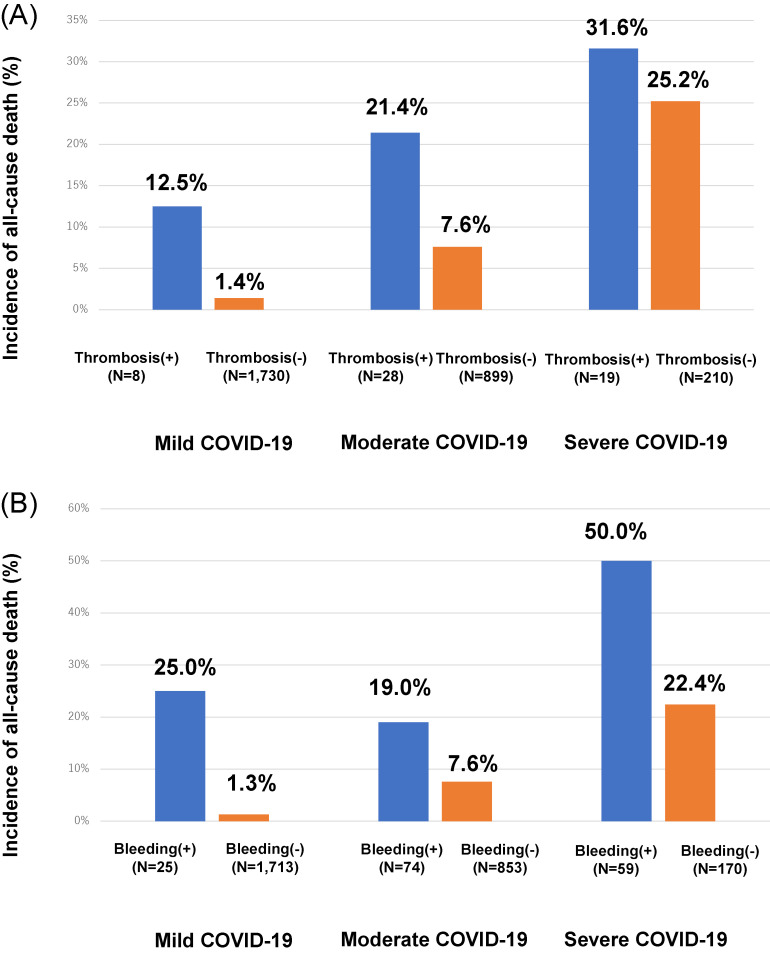
Comparative incidences of thrombosis (A) and major bleeding (B) between the groups of patients who died and those who survived in analyses that were stratified by the COVID-19 severity on admission. COVID-19, novel coronavirus disease 2019.

In patients who died, the incidence of thrombosis was higher than in those who survived (8.2% vs 1.5%, *P* < 0.001; Table 2). Similarly, in patients who developed thrombosis, the mortality rate was numerically higher than that in those who did not develop thrombosis, regardless of the COVID-19 severity on admission (mild, 12.5% vs 1.4%; moderate, 21.4% vs 7.6%; severe, 31.6% vs 25.2%; Figure 2A).

**Table 2.** Clinical outcomes during hospitalization

	Total (*N* = 2,894)	Patients who died during hospitalization (*N* = 158)	Patients alive at discharge (*N* = 2,736)	*P*-value
Thrombosis	55 (1.9%)	13 (8.2%)	42 (1.5%)	<0.001
VTE	39 (1.3%)	8 (5.1%)	31 (1.1%)	0.001
Arterial thrombotic events	12 (0.4%)	3 (1.9%)	9 (0.3%)	<0.001
Ischemic stroke	9 (0.3%)	2/3 (67%)	7/9 (78%)	—
Myocardial infarction	2 (0.07%)	1/3 (33%)	1/9 (11%)	—
Systemic arterial thromboembolism	1 (0.04%)	0/3 (0%)	1/9 (11%)	—
Other thrombosis	7 (0.2%)	3 (1.9%)	4 (0.1%)	<0.001
Major bleeding	57 (2.0%)	20 (12.7%)	37 (1.4%)	<0.001

VTE, venous thromboembolism.

The clinical outcomes are presented as numbers of events and percentages, which were compared using the chi-squared test when appropriate; otherwise, Fisher’s exact test was used as categorial variables.

In patients who died, the incidence of major bleeding was higher than that in those who survived (12.7% vs 1.4%, *P* < 0.001; Table 2). Similarly, the mortality was higher in patients who developed bleeding than in those who did not develop thrombosis, regardless of the COVID-19 severity on admission (mild, 25.0% vs 1.3%; moderate, 19.0% vs 7.6%; severe, 50.0% vs 22.4%; Figure 2B).

### Risk factors of all-cause mortality during hospitalization

**Figure 3.**
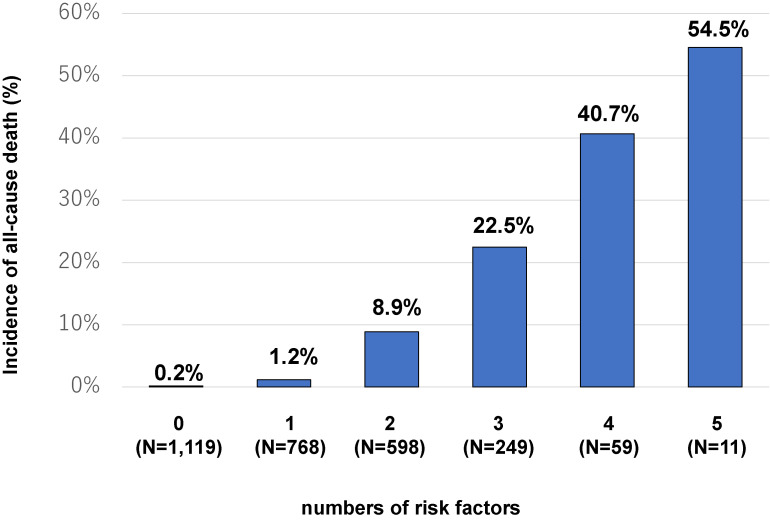
Incidences of all-cause mortality according to the number of potential mortality-associated risk factors, including age >70 years, high D-dimer values on admission, heart disease, active cancer, more severe COVID-19 on admission, and onset of major bleeding during hospitalization. COVID-19, novel coronavirus disease 2019.

We performed multivariate logistic regression analysis and revealed that age >70 years; D-dimer >1.0 µg/mL on admission; heart disease, active cancer, or more severe COVID-19 on admission; and major bleeding during hospitalization were independently associated with a higher mortality risk (Table 3). Among the potential risk factors for mortality, severe COVID-19 on admission had the strongest influence on mortality (adjusted OR 13.20; 95% CI, 6.72–26.10), followed by age >70 years (adjusted OR 7.96; 95% CI, 5.05–12.60). A higher number of potential risk factors for mortality was linked to a higher incidence of all-cause mortality (Figure 3).

**Table 3.** Multivariable analysis for the risk factors for all-cause mortality during hospitalization

	Univariate		Multivariable	
	
	OR (95% CI)	*P*-value	OR (95% CI)	*P*-value
Age >70 years	8.85 (6.31–12.40)	<0.001	7.96 (5.05–12.60)	<0.001
Men	1.20 (0.85–1.70)	0.30	1.02 (0.67–1.55)	0.92
Body mass index ≥30 kg/m^2^	0.76 (0.47–1.23)	0.26	1.18 (0.65–2.14)	0.59
D-dimer levels on admission >1.0 µg/mL	8.41 (5.59–12.70)	<0.001	2.90 (1.83–4.59)	<0.001
Hypertension	3.21 (2.32–4.44)	<0.001	1.28 (0.84–1.92)	0.25
Diabetes mellitus	1.68 (1.18–2.40)	0.003	0.95 (0.61–1.46)	0.80
Heart disease	3.97 (2.71–5.84)	<0.001	1.69 (1.03–2.76)	0.04
Respiratory disease	1.36 (0.85–2.19)	0.20	0.76 (0.42–1.38)	0.37
Active cancer	5.13 (2.71–9.69)	<0.001	3.20 (1.44–7.09)	0.004
History of major bleeding	2.94 (1.01–8.56)	0.049	1.30 (0.38–4.45)	0.67
History of VTE	4.39 (1.23–15.70)	0.02	1.39 (0.25–7.75)	0.71
Severity of COVID-19 on admission				
Mild (Reference)	—	—	—	—
Moderate (versus Mild)	5.94 (3.75–9.42)	<0.001	3.02 (1.74–5.25)	<0.001
Severe (versus Mild)	23.80 (14.50–39.00)	<0.001	13.20 (6.72–26.10)	<0.001
Pharmacological thromboprophylaxis	6.75 (4.46–10.20)	<0.001	1.46 (0.86–2.50)	0.16
Development of thrombosis during hospitalization	5.75 (3.02–11.00)	<0.001	1.77 (0.78–3.99)	0.17
Development of major bleeding during hospitalization	10.60 (5.98–18.70)	<0.001	2.44 (1.17–5.05)	0.02

CI, confidence interval; COVID-19, novel coronavirus disease 2019; OR, odds ratio; VTE, venous thromboembolism.

Adjusted for age >70 years, male sex, body mass index >30 kg/m^2^, D-dimer values on admission >1.0 µg/mL, hypertension, diabetes mellitus, heart disease, respiratory disease, active cancer, history of major bleeding, history of VTE, and moderate and high COVID-19 severity at admission.

## DISCUSSION

The study’s main findings are that, compared to those who survived to discharge, patients who died: i) were older, had higher D-dimer values on admission, and had more comorbidities; ii) had higher incidences of thrombosis and major bleeding during hospitalization; and iii) had independent risk factors for mortality, including age >70 years, D-dimer >1.0 µg/mL on admission, heart disease, active cancer, more severe status of COVID-19 on admission, and major bleeding following hospitalization.

Several previous reports of the risk factors for the disease severity and progression of COVID-19 in Japan have been published.^31^^,^^32^ A recent large-scale Japanese registry-based study that included 3,829 COVID-19 patients reported that male sex, higher age, hypertension, obesity, cardiovascular disease, and diabetes were independent risk factors for disease progression.^17^ However, the mortality-associated risk factors for COVID-19 patients in Japan have not been reported. A large-scale United Kingdom cohort study that examined the risk factors for mortality in more than 17 million COVID-19 patients showed that higher age, poverty, male sex, liver disease, diabetes, asthma, and kidney disorders were independent risk factors for mortality.^33^ Another population-based large observational study that evaluated data from 1.8 million people in Stockholm, Sweden, reported that heart failure, ischemic heart disease, obesity, kidney failure, and diabetes were independent risk factors for mortality.^28^ A recent meta-analysis of 42 studies in 423,117 COVID-19 patients revealed the following independent risk factors for mortality: higher age, male sex, current smoking, chronic obstructive pulmonary disease, acute kidney injury, active cancer, diabetes, heart disease, obesity, hypertension, and a high D-dimer value on admission.^15^ Our study revealed that, in Japan, old age, heart disease, active cancer, and high D-dimer values on admission were mortality-related risk factors in COVID-19 patients. Furthermore, this study showed that severe COVID-19 on admission had the most substantial influence on mortality, which is in line with previous studies that reported severe COVID-19 as a strong risk factor for severe respiratory conditions and the use of invasive mechanical ventilation.^14^

Several risk factors for mortality are in line with those reported previously, while some are not. COVID-19 mortality risk differs among races; therefore, the risk factors for mortality might vary depending on the race.^6^^,^^34^^–^^36^ The overall mortality rate for COVID-19 in Japan was 0.34% as of June 2022,^37^ which is approximately one fourth the global mortality rate (1.2%).^38^ A meta-analysis of data from more than 423,000 patients in countries other than Japan showed an in-hospital mortality rate of 17.6%.^15^ In this study, the in-hospital mortality rate was 5.5%, which is consistent with that reported in another Japanese registry-based study of 5,194 patients with COVID-19 (5.7%).^39^ The reasons for the differences in mortality and the risk factors for mortality in COVID-19 patients remain unclear; however, some possible mechanisms have been speculated. First, COVID-19 mortality is affected by not only patient characteristics but also the socioeconomic status and quality of clinical care and medical service.^36^ Second, different patient characteristics, including the prevalence of obesity and comorbidities, depending on the country, could have had a specific influence on the mortality risk.^40^ Third, different genetic properties, such as human leukocyte antigen,^41^^–^^44^ angiotensin-converting enzyme 1 polymorphism,^45^ Neanderthal haplotypes,^46^ and vitamin D^47^ could explain the racial differences in mortality. Fourth, depending on each country, various hospitalization options could have influenced the in-hospital mortality. Sixth, the hospitalization criteria in each facility could vary according to the community and affect the in-hospital mortality. Seventh, virus variants could affect mortality; however, the mortality rate was 5.5% in the present study, which was similar to that of a previous study that was conducted during the first and second waves (5.7%).^48^

Notably, thrombosis was not identified as an independent risk factor for COVID-19-associated mortality in this study, contrary to the findings reported previously.^49^^–^^51^ Several studies have shown that thrombosis during hospitalization influences COVID-19-related mortality. Compared to those without VTE, patients with VTE have a higher mortality risk.^49^ The present study revealed that the development of thrombosis during hospitalization had a numerically high OR of 1.77 for mortality, although this was not statistically significant. This association can be partly explained as follows. The lower incidence of thrombosis in Japan relative to other countries could explain the reduced statistical power for detecting significant differences. The incidence of thrombosis in this study was 1.9%, whereas the incidence in another study that analyzed data from Europe, the United States, and China showed an incidence of 21%, although the absolute OR of thrombosis for mortality was 1.74,^50^ which might be consistent with the result obtained in the present study.

Obesity is reported as a mortality-related risk factor in several studies that included patients with COVID-19 worldwide. A meta-analysis that examined obesity as a risk factor for mortality in patients with COVID-19 overseas reported a risk ratio of 1.42 (95% CI, 1.24–1.63),^51^ while a national study in Japan revealed that Japanese COVID-19 patients with a high BMI showed a higher risk for progression of disease severity, although this was not associated with a higher risk for mortality.^52^ In line with a previous study, we showed that obesity in individuals with a BMI ≥30 kg/m^2^ was not an independent risk factor associated with lower mortality risk. Specifically, Japanese patients commonly have lower body weights than those of other ethnicities, and low body weight is reportedly associated with mortality risk^53^; the body weights in Japan versus overseas could affect this difference in these results.

There are several limitations of this study. First, the observational study design inherently confers various biases. In particular, the attending physicians made all treatment-related decisions, which could have influenced the clinical results. Second, only the clinical outcomes during hospitalization were analyzed in this study; therefore, the risk of postdischarge mortality could not be ascertained. Third, although we tried to comprehensively explore the potential risk factors for mortality, there could be other unknown risk factors that were not considered in the present study. Fourth, there were no data regarding the treatment during hospitalization and vaccination status, which might have affected mortality.

In conclusion, this large-scale observational study in Japan identified several independent risk factors for mortality in hospitalized COVID-19 patients that could facilitate appropriate risk stratification of COVID-19 patients.

## SUPPLEMENTARY MATERIAL

eMaterial 1. Definitions of patient characteristics
