# Supplementary material for: Mortality-associated Risk Factors in Hospitalized COVID-19 Patients in Japan: Findings of the CLOT-COVID Study
Source: J Epidemiol. 2023 Mar 5;33(3):150–7. doi: 10.2188/jea.JE20220201 (PMC9909175; doi:10.2188/jea.JE20220201)
Supplement: Supplementary file 1 [file je-33-150-s001.pdf]

eMaterial 1. Definitions of patient characteristics

Hypertension was diagnosed if the peripheral blood pressure was >140/90 mm Hg or if the patient was taking medication for hypertension. The presence of diabetes was diagnosed using the hemoglobin A1c (HbA1c) (National Glycohemoglobin Standardization Program, 6.5%) as the standard or was assumed if the patient was taking medication for the treatment of diabetes. Heart disease was defined as heart disorders such as heart failure, angina pectoris, and a history of myocardial infarction. Heart failure was diagnosed if the patient had a history of hospitalization for heart failure, the patient had symptoms due to heart failure (New York Heart Association functional class ≥2), or the left ventricular ejection fraction was <40%. Respiratory disease was defined as a persistent lung disorder such as asthma, chronic obstructive pulmonary disease, or restrictive lung disease. Patients with active cancer were defined as those on treatment for cancer, such as chemotherapy or radiotherapy, those scheduled to undergo cancer surgery, those with metastasis to other organs, and/or those with terminal cancer.[1] A history of major bleeding was diagnosed if the patient had a history of International Society of Thrombosis and Hemostasis major bleeding, which consisted of a reduction in the hemoglobin level by at least 2 g/dL, transfusion of at least two units of blood, or symptomatic bleeding in a critical area or organ.[2]

REFERENCES

1. Sakamoto J, Yamashita Y, Morimoto T, Amano H, Takase T, Hiramori S, Kim K, Oi M, Akao M, Kobayashi Y, Toyofuku M, Izumi T, Tada T, Chen PM, Murata K, Tsuyuki Y, Saga S, Nishimoto Y, Sasa T, Kinoshita M, Togi K, Mabuchi H, Takabayashi K, Yoshikawa Y, Shiomi H, Kato T, Makiyama T, Ono K, Tamura T, Nakagawa Y, Kimura T; COMMAND VTE Registry Investigators. Cancer-Associated Venous Thromboembolism in the Real World - From the COMMAND VTE Registry. Circ J. 2019;83(11):2271-2281.

2. Schulman S, Kearon C, Subcommittee on Control of Anticoagulation of the Scientific and Standardization Committee of the International Society on Thrombosis and Haemostasis. Definition of major bleeding in clinical investigations of antihemostatic medicinal products in non-surgical patients. J Thromb Haemost 2005;3:692–694
